# Localization and Actuation for MNPs Based on Magnetic Field-Free Point: Feasibility of Movable Electromagnetic Actuations

**DOI:** 10.3390/mi11111020

**Published:** 2020-11-21

**Authors:** Chan Kim, Jayoung Kim, Jong-Oh Park, Eunpyo Choi, Chang-Sei Kim

**Affiliations:** 1 School of Mechanical Engineering, Chonnam National University, Gwangju 61186, Korea; kc9134@gmail.com (C.K.); eunpyochoi@jnu.ac.kr (E.C.); 2 Korea Institute of Medical Microrobotics, Gwangju 61011, Korea

**Keywords:** movable electromagnetic actuation system, field-free point, 3D localization

## Abstract

Targeted drug delivery (TDD) based on magnetic nanoparticles (MNPs) and external magnetic actuation is a promising drug delivery technology compared to conventional treatments usually utilized in cancer therapy. However, the implementation of a TDD system at a clinical site based on considerations for the actual size of the human body requires a simplified structure capable of both external actuation and localization. To address these requirements, we propose a novel approach to localize drug carriers containing MNPs by manipulating the field-free point (FFP) mechanism in the principal magnetic field. To this end, we devise a versatile electromagnetic actuation (EMA) system for FFP generation based on four coils affixed to a movable frame. By the Biot–Savart law, the FFP can be manipulated by appropriately controlling the gradient field strength at the target area using the EMA system. Further, weighted-norm solutions are utilized to correct the positions of FFP to improve the accuracy of FFP displacement in the region of interest (ROI). As MNPs, ferrofluid is used to experiment with 2D and 3D localizations in a blocked phantom placed in the designed ROI. The resultant root mean square error of the localizations is observed to be approximately 1.4 mm in the 2D case and 1.6 mm in the 3D case. Further, the proposed movable EMA is verified to be capable of simultaneously scanning multiple points as well as the actuation and imaging of MNPs. Based on the success of the experiments in this study, further research is intended to be conducted in scale-up system development to design precise TDD systems at clinical sites.

## 1. Introduction

Targeted drug delivery (TDD) is considered to be a competitive solution to the treatment requirements for cancerous tumors. TDD has exhibited the advantages of reduced toxicity and dose optimization, which were the primary issues in existing cancer therapies, including radiotherapy, chemotherapy, and hormone therapy [1]. Several material candidates have been studied in the context of the implementation of TDD. Magnetic nanoparticles (MNPs) have emerged as the most promising carrier composition for TDD, owing to their magnetic properties that can be utilized to dislocate them using magnetic fields induced by external devices [2]. Their nano-scale and ability to fabricate various shapes with surface coating materials are theoretically expected to make them even more suitable as the primary material for TDD [3]. However, in practice, aggregated particles in a vascular environment are difficult to maneuver as their small scale, in conjunction with the lack of nano-particle imaging, degrades the efficiency of nanoparticle steering [4].

Electromagnetic actuators (EMAs) are widely utilized to steer magnetic drug carriers to targeted lesions [5], as well as micro- and millimeter-scaled robot systems, such as capsule endoscopes (CEs) [6,7]. Other approaches, such as acoustic radiation force (ARF), bilayer-structured microrobots reacting thermo-electromagnetically, and octagram-shaped micro grippers, have also been researched [8,9]. Most drug delivery mechanisms based on MNPs are steered to target sites by applying an external magnetic field that interacts with the MNPs. The critical barrier of nanoparticle systems is the generation of adequately high magnetic and gradient fields to manipulate MNPs. In this context, the magnetic resonance navigation (MRN) system was devised based on magnetic resonance imaging (MRI) to steer and capture images of nanoparticles simultaneously using electromagnetic coils [10]. MRI is already clinically available and it can provide much better resolution and contrast of images in wide ranges along with independence of imaging points. In vivo investigations of the MRN system were successfully conducted in a carotid artery of a living pig with a 1.5 mm diameter magnetic bead [11]. However, TDD application to nano-scale particles has not been fully addressed yet, so a new approach capable of both guiding and imaging magnetic nanoparticles needs to be considered [12].

Alternatively, an MNP-tracking system known as magnetic particle imaging (MPI) was introduced by Gleich and Weizenecker in 2005 [13]. This medical imaging modality has been studied recently in the context of direct measurement of the aggregation of magnetic nanoparticles [14]. Compared to other imaging methodologies, MPI is capable of realizing higher spatial and temporal resolutions owing to its enhanced sensitivity in the detection of superparamagnetic iron oxides (SPIOs), which have been clinically demonstrated to be a contrast agent material utilized in MRI [15]. Its utilization of SPIOs is one of the most important features of MPI, as it enables a safer testing environment compared to other angiographies, such as X-ray, magnetic resonance, and computed tomography, whose contrast agents include iodine and, therefore, may pose health risks to patients, especially those with chronic kidney disease (CKD) [16].

As MPI is capable of selectively detecting the reflected signals from MNPs, it can derive more distinct images of MNPs compared to other methods. Its fundamental principle is to obtain nonlinear responses of SPIOs induced by the exposure of specified areas where the value of the magnetic field is zero, i.e., field-free points (FFPs) and field-free lines (FFLs) [17]. Previous studies have utilized four permanent magnetics arranged in the shape of a quadrupole to achieve static FFPs [18]. The MPI hardware uses a Halbach array [19] to generate FFPs and FFLs by manipulating the angle between adjacent permanent magnets [20]. The traditional magnetic arrangements mentioned above require additional devices to drive the FFP along a trajectory because it is a static field [13]. A mechanical device for movement of the coil system or the scanned object was applied to the coil system [21]. However, the disadvantage of this method causes a lack of speed for scanning, so supportive drive coils generating the homogenous magnetic fields to push away a static FFP to the designated position were set up in an MPI scanner [15]. However, the equipped MPI scanner has a spatial limitation, because at least three pairs of drive coils need to be set up to manipulate FFPs in three-dimensional space [13]. Future studies should investigate the influence of spatial interference on electromagnetically induced FFP movement in human-sized MPI devices.

As solutions of FFP and FFL generation, two major categories of image reconstruction based on signals reflected from nanoparticles—harmonic-space MPI and X-space MPI—have been extensively researched [22]. Harmonic-space MPI is based on a system matrix comprising Fourier-transformed signals generated by the translation of FFPs along the designated path [22]. Any rearrangement of the transformed data within the system matrix necessitates reconstruction via matrix inversion [15]. This becomes especially complicated with an increase in the size of the system matrix [22,23,24]. In contrast, X-space MPI is a simplified method that does not require matrix inversion and yet enjoys multiple strengths [23]. It directly utilizes the prompt MPI signal and velocity of FFPs in the field of view (FOV) to reduce the computational duration required to reconstruct the image [24]. Spatial resolutions of 1.7 mm have been successfully measured in the literature using tailored MPI tracers (UW-1) featuring a core diameter of 26–27 mm and a hydrodynamic of ~50 nm or ~72 nm for qualified imaging [25].

Besides imaging, the FFPs and FFLs generated by EMAs are capable of steering and manipulating nanoparticles. Nothnagel et al. demonstrated this property by using six tempered soft magnetic spheres for manipulation and two orthogonal soft magnetic needles for the acquisition of localization signals by varying the FFP position [26]. Bakenecker et al. implemented a combined system for actuation and imaging based on a savory-shaped swimmer model filled with a mixture of acrylic paint and Perimag nanoparticles within the Y-shaped vessel phantom [27]. However, scaled-up variants of such systems meant for clinical applications require a large amount of space for the MPI equipment, such as the MPI scanner [27], and thus require further refinement.

In this context, we propose a novel approach for the actuation and imaging of MNPs in 3D regions of interest (ROIs) with the goal of developing a precise drug delivery system. The proposed method is based on a movable frame of an EMA system that can control electromagnetic field to generate FFPs. By virtue of the movable EMA configuration, the system size and the number of constituent coils can be minimized and the workspace can be lengthened in the direction of movement during the localization and actuation of nanoparticles in the ROI. The proposed EMA design produces an extensive procedure space that induces a gradient field oriented in the direction opposite to that within the actuation coils while actuating the FFP within the coil system. Ultimately, the two disparate directions of magnetic gradient fields—one outside the coil and the other inside—generate a 1D trapping point capable of steering the swarm of nanoparticles.

The primary contribution of this paper is the successful development of a movable EMA and a control method for the imaging and steering of MNPs in 3D space. Moreover, the proposed method was validated via a sequential procedure in a bifurcation phantom. Compared to the conventional MNP-steering approaches based on MPI scanners introduced in [26,27], the proposed architecture enables the implementation of magnetic tracers using fewer coils, making it a more efficient actuation method in TDD systems. The proposed method has the potential of practical clinical implementation due to its minimal installation space and easy access to patients during the procedure.

## 2. Methods

### 2.1. System Description

**Figure 1:**
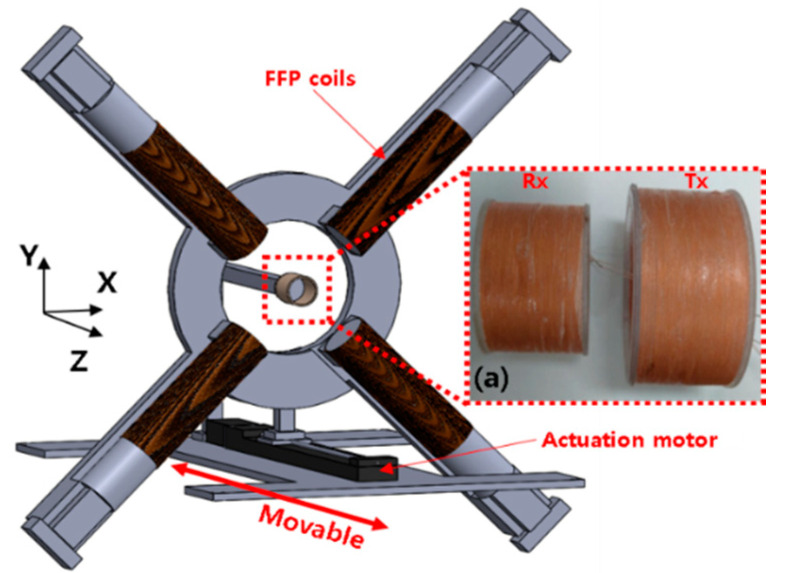
The configuration of the proposed movable system for magnetic particle imaging (MPI) based on four coils. The mainframe of the system can be moved one-dimensionally by the actuation motor to scan the Z-axis. (**a**) To receive the signals reflected by the particles, a combined coil comprising a transmitting coil and a receiver coil was installed at the center of the region of interest (ROI).

Spatial resolution is an important metric for the performance of MPI. [28]. Two governing factors of MPI are gradient field value and the core size of the particle [29]. In this paper, the latter is neglected since this study is focused on the FFP generation method. Based on the aforementioned imaging qualification criterion introduced in previous research, most MPI systems require more than six electromagnets to generate 3D FFPs with appropriate gradient fields for spatial imaging of MNPs. In this paper, we reduced the number of required coils to four during the design of the movable EMA system and achieved concurrent imaging and actuation by utilizing FFPs, as depicted in Figure 1.

First, the position of the aggregated MNP along the Z-axis is identified by measuring the reflected magnetic field intensity while appropriately moving the EMA system. Once the position of maximum intensity is determined, the XY-planar image is obtained by scanning the surface using FFP control. Finally, the 3D position of the aggregated MNP is reconstructed by combining the longitudinal movable (z-direction) position and the scanned results of the XY-planar image.

### 2.2. FFP Generation

The FFP denotes a point in the magnetic field where the magnetic force is zero. The magnetic force (F) exerted on the surface of a material can be expressed as follows.

(1) F=V(M·∇)B

where M denotes the magnetization value of the magnetic object and ∇ denotes the gradient. The general equation relating magnetic flux, B, with magnetic flux intensity, H, of which the unit is A/m, is as follows.

(2) $$B=\mu_{0}\mu_{r}H$$

where $\mu_{r}$ denotes the permeability of the material. The magnetic flux density, H, can be derived from the electric current transmitted through the electric wires of the electromagnets via the Biot–Savart law. The magnetic force can be expressed in terms of its components along each spatial direction as:

(3) $$F=V\left[\begin{array}{ccc} \frac{\partial B_{x}}{\partial x} & \frac{\partial B_{y}}{\partial x} & \frac{\partial B_{z}}{\partial x}\\ \frac{\partial B_{x}}{\partial y} & \frac{\partial B_{y}}{\partial y} & \frac{\partial B_{z}}{\partial y}\\ \frac{\partial B_{x}}{\partial z} & \frac{\partial B_{y}}{\partial z} & \frac{\partial B_{z}}{\partial z} \end{array}\right]\left[\begin{array}{c} M_{x}\\ M_{y}\\ M_{z} \end{array}\right]$$

Since the magnetic flux density, B, which determines the FFP at the desired point, is a controllable parameter in (1), we first defined B of one coil in the system that generates the magnetic field at the desired position (*x, y, z*) in the region of interest in the EMA as follows, where the applied current is $I={\left[i_{1}\;i_{2}\;i_{3}\;i_{4}\right]}^{T}$.

(4) $$B\left(x,y,z\right)=\hat{B}\left(x,y,z\right)I$$

By utilizing the superposition property of magnetic fields for the four fields induced by the four coils in this paper, the magnetic flux density at (x,y,z) can be expressed as follows.

(5) $$B(x,y,z)=\left[\begin{array}{c} B_{x}\\ B_{y}\\ B_{z} \end{array}\right]\left[\begin{array}{cc} {\hat{B}}_{x,1}\left(P\right) & {\hat{B}}_{x,2}\left(P\right)\;\;\;\\ {\hat{B}}_{y,1}\left(P\right) & {\hat{B}}_{y,2}\left(P\right)\;\;\;\\ {\hat{B}}_{z,1}\left(P\right) & {\hat{B}}_{z,2}\left(P\right)\;\;\; \end{array}\begin{array}{cc} {\hat{B}}_{x,3}\left(P\right) & {\hat{B}}_{x,4}\left(P\right)\\ {\hat{B}}_{y,3}\left(P\right) & {\hat{B}}_{y,4}\left(P\right)\\ {\hat{B}}_{z,3}\left(P\right) & {\hat{B}}_{z,4}\left(P\right) \end{array}\right]\left[\begin{array}{c} i_{1}\\ i_{2}\\ \begin{array}{c} i_{3}\\ i_{4} \end{array} \end{array}\right]=\left[\begin{array}{c} {\hat{B}}_{x}\left(P\right)\\ {\hat{B}}_{y}\left(P\right)\\ {\hat{B}}_{z}\left(P\right) \end{array}\right]I$$

where P=(x,y,z) denotes the location of the calculated point. Further, following the same method as that used to express magnetic flux density by Equation (3), the magnetic force can be expressed in terms of the partial derivatives in each direction as follows.

(6) $$\frac{\partial B\left(P\right)}{\partial x}=\left[\frac{\partial {\hat{B}}_{1}\left(P\right)}{\partial x}\;\;\frac{\partial {\hat{B}}_{2}\left(P\right)}{\partial x}\;\;\frac{\partial {\hat{B}}_{3}\left(P\right)}{\partial x}\;\frac{\partial {\hat{B}}_{4}\left(P\right)}{\partial x}\right]\left[\begin{array}{c} i_{1}\\ i_{2}\\ \begin{array}{c} i_{3}\\ i_{4} \end{array} \end{array}\right]=\frac{\partial \hat{B}\left(P\right)}{\partial x}I\;\frac{\partial B\left(P\right)}{\partial y}=\left[\frac{\partial {\hat{B}}_{1}\left(P\right)}{\partial y}\;\;\frac{\partial {\hat{B}}_{2}\left(P\right)}{\partial y}\;\;\frac{\partial {\hat{B}}_{3}\left(P\right)}{\partial y}\;\frac{\partial {\hat{B}}_{4}\left(P\right)}{\partial y}\right]\left[\begin{array}{c} i_{1}\\ i_{2}\\ \begin{array}{c} i_{3}\\ i_{4} \end{array} \end{array}\right]=\frac{\partial \hat{B}\left(P\right)}{\partial y}I\;\frac{\partial B\left(P\right)}{\partial z}=\left[\frac{\partial {\hat{B}}_{1}\left(P\right)}{\partial z}\;\;\frac{\partial {\hat{B}}_{2}\left(P\right)}{\partial z}\;\;\frac{\partial {\hat{B}}_{3}\left(P\right)}{\partial z}\;\frac{\partial {\hat{B}}_{4}\left(P\right)}{\partial z}\right]\left[\begin{array}{c} i_{1}\\ i_{2}\\ \begin{array}{c} i_{3}\\ i_{4} \end{array} \end{array}\right]=\frac{\partial \hat{B}\left(P\right)}{\partial z}I$$

**Figure 2:**
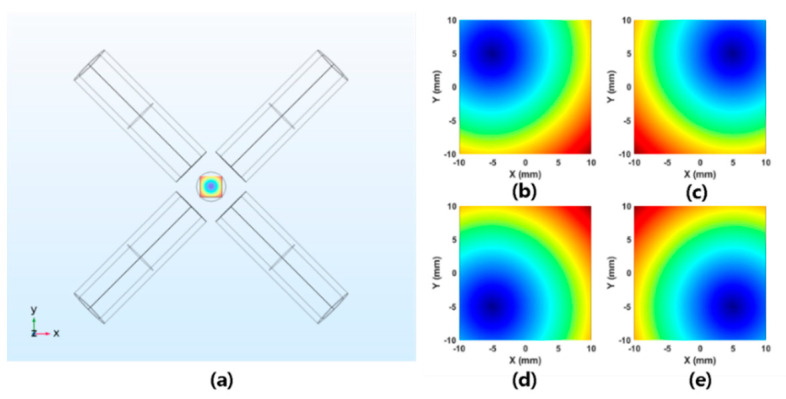
The simulated field-free point (FFP) obtained from COMSOL on the XY-plane. (**a**) A simulation model of the proposed system. (**b**–**e**) The flexible mobility of the proposed FFP actuation structure in the system. The FFP is located at (−5,5) in (**b**), (5,5) in (**c**), (−5,−5) in (**d**), and (5,−5) in (**e**).

The gradient terms derived in (6) can be used to express Equation (1) of magnetic force in the following form.

(7) $$F=V{\left[\frac{\partial B\left(P\right)}{\partial x}\;\frac{\partial B\left(P\right)}{\partial y}\;\frac{\partial B\left(P\right)}{\partial z}\right]}^{T}M=V\left[\begin{array}{c} M^{T}\frac{\partial \hat{B}\left(P\right)}{\partial x}\\ M^{T}\frac{\partial \hat{B}\left(P\right)}{\partial y}\\ M^{T}\frac{\partial \hat{B}\left(P\right)}{\partial z} \end{array}\right]I$$

where $M={\left[\;M_{x}\;M_{y}\;M_{z}\;\right]}^{T}$. Now, the two governing equations, (3) and (7), are combined to calculate the magnetic field in the ROI. Thus, the primary equation is expressed as follows.

(8) $$D=\left[\begin{array}{c} B\left(P\right)\\ M^{T}G_{x}\left(P\right)\\ \begin{array}{c} M^{T}G_{y}\left(P\right)\\ M^{T}G_{z}\left(P\right) \end{array} \end{array}\right]\left[\begin{array}{c} i_{1}\\ i_{2}\\ \begin{array}{c} i_{3}\\ i_{4} \end{array} \end{array}\right]=M_{u}\left(P\right)I$$

where $D={\left[B\;F\right]}^{T}$ denotes the desired matrix and $M_{u}\in R^{12\times 4}$ denotes the unit matrix corresponding to the four coils in the range of the ROI. To obtain the value of the input current in (8), matrix inversion is applied to $M_{u}$, and a pseudo inversion is applied to obtain the current matrix via the following equation in analogy with the methodology of [30].

(9) $$I=M_{u}{}^{+}D$$

where $M_{u}{}^{+}$ denotes the pseudo inverse matrix of $M_{u}$ that is obtained via a simulation from COMSOL Multiphysics (COMSOL Group, Sweden) based on the same physical conditions of coils, including the number of turns and scales, as those depicted in Figure 2a. Since COMSOL is normally utilized for magnetic field analysis based on the finite element method, an accurate and reliable solution could be obtained. The ROI of our system is taken to be 20 × 20 ${\mathrm{mm}}^{2}$. We assume the interval distance of FFPs along each axis to be 1 mm. Therefore, a total of 441 entities of the unit matrix were collected through the basic data calculation for the current value. The dependence expressed by Equation (9) can be utilized to manipulate the magnetic torque and force within the field of control of a microrobot’s movement in the ROI. To use this strategy to induce an FFP at a pre-determined point, the entries of B(P) related to the desired matrix in (10) are set to zero, which can capture the domination of particle signals by the gradient strength value of the given nonlinear magnetization curve. Then, by using Equation (8), the desired matrix can be expressed as follows.

(10) $$D={\left[B_{x}\;B_{y}\;B_{z}\;G_{xx}\;G_{xy}\;G_{xz}\;G_{yx}\;G_{yy}\;G_{yz}\;G_{zx}G_{zy}\;G_{zz}\;\right]}^{T}$$

where the values of $B_{x}$, $B_{y}$, and $B_{z}$ are assumed to be zero. The additional theoretical support to manipulate the FFP is obtained from Maxwell’s equations with the constraint of $G_{xx}+G_{yy}+\;G_{zz}=0$.

In our case, $G_{xx}$, $G_{yy}$, and $G_{zz}$ can be substituted with $-\alpha G_{zz}$, $\left(\alpha -1\right)G_{zz}$, and $G_{zz}$, where α∈(0,1) [31]. Further, α is set to 1/2. The simulation results of FFP control based on the proposed magnetic field computations are depicted in Figure 2.

(11) ∇·B=0

### 2.3. Weighted-Norm Method

We simulate the FFP control method at all points within the ROI to evaluate the accuracy of the proposed method. However, the desired FFP does not correspond closely with the simulated FFP locations corresponding to several points within the ROI. Further, substitution of the current values obtained via (9) into (8) to verify the vanishing of the magnetic field value reveals that $B_{x}$, $B_{y}$, and $B_{z}$ remain non-zero. To prevent this mislocation of FFPs within the ROI, we apply weighted-norm solutions [32] based on least-squares solutions to minimize the error norm in the associated linear equation. Under this scheme, $I\in R^{n}$

(12) $$I=Q_{0}{}^{-1}M_{u}{}^{+}D$$

where $M_{u}\in R^{m\times n}$, $D\in R^{m},\;\mathrm{and}\;Q_{0\;}\in R^{m\times m}$, which is the square root of $Q_{\;}$ utilized for the configuration of the weighted norm. Therefore, $Q_{0\;}$ and Q are related by the following equation.

(13) $$Q=Q_{0}{}^{T}Q_{0}$$

To construct the weighted diagonal matrix, $Q_{0}{}^{-1}$, we add the diagonal matrix, $W_{dig}\in R^{3\times 3}$, including $W_{1}$, $W_{2}$, and $W_{3}$, to the constraints, $B_{x}$, $B_{y}$, and $B_{z}$, as follows.

(14) $$W_{dig}=\left[\begin{array}{ccc} W_{1} & 0 & 0\\ 0 & W_{2} & 0\\ 0 & 0 & W_{3} \end{array}\right]$$

where the completed form of $Q_{0}{}^{-1}\in R^{12\times 12}$ is given by

(15) $$Q_{0}{}^{-1}=\left[\begin{array}{cc} W_{dig} & 0\\ 0 & I_{dig} \end{array}\right]$$

where $I_{dig}\in R^{9\times 9}$. We increase the value of the weight components in $W_{dig}$ to identify the minimum value required to obtain an FFP at the exact desired site in order to evaluate the performance of the proposed method based on weighted-norm solutions. During this evaluation, we assume all weight variables in the weight matrix share the same value. Table 1 shows the observed distance errors in the case of an FFP positioned at (8,0) within the ROI.

**Figure 3:**
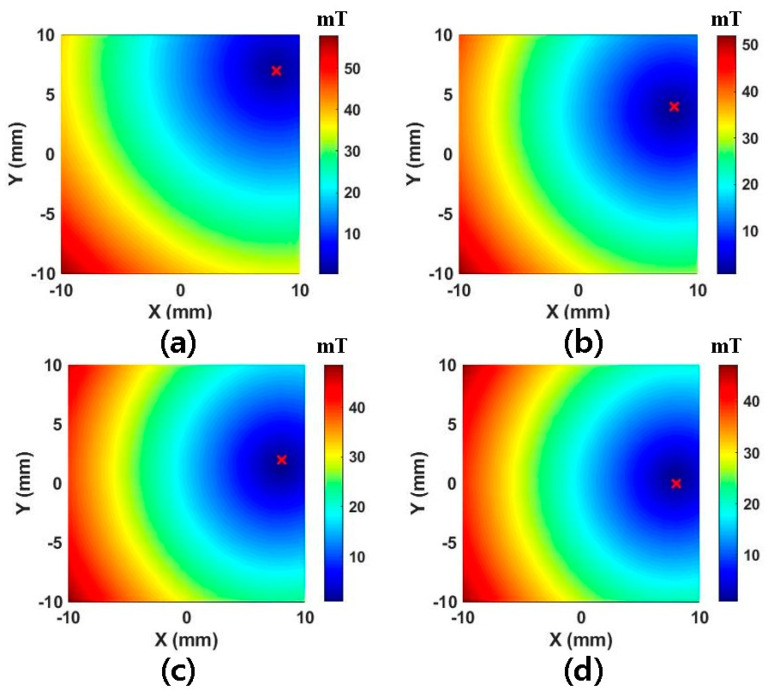
The desired FFP location is assumed to be (8,0). (**a**) The FFP field mapping corresponding to a weight of 30. (**b**) The FFP field mapping corresponding to a weight 50. (**c**) The FFP field mapping corresponding to a weight of 90. (**d**) The FFP field mapping corresponding to a weight of 141. The position corresponding to the minimum value (red mark in each field map) is at (8,7) in (**a**), (8,4) in (**b**), (8,2) in (**c**), and (8,0) in (**d**).

As is evident from the table, 141 points is the minimum value for FFP actuation. Figure 3 depicts the simulated magnetic field map corresponding to Table 1, in which the weight value is taken to be 30 in (a), 50 in (b), 90 in (c), and 141 in (d). Even if 141 points are selected, the FFP location is observed to be inaccurate at the edge of the ROI. To remedy this, we increase the number to 1000, which ensures the accurate position of FFPs over the entire ROI. Finally, it should be noted that the aforementioned method can be applied to any electromagnetic system, irrespective of the structure of its coils, for the actuation of FFPs in 3D space.

## 3. Prototyping

**Figure 4:**
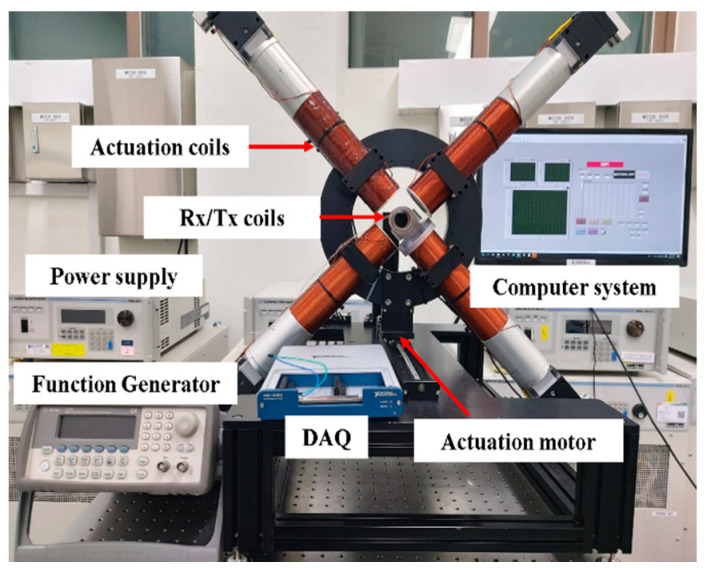
The four-coil scanning system.

The proposed system is depicted in Figure 4. Each coil producing a magnetic field is situated at a distance of 40 mm from the center of the coordinate system. Pure iron cores, each with a diameter of 21 mm, are covered by winding solenoid coils to maximize eventual FFP control. The core type design is based on the fact that the iron-cored MPI system exhibits a lower current value by more than 60% for each coil compared to air-cored system, besides producing higher resolution image [33].

The prototyped system comprises two pairs of coils to make it amenable to future studies of open-type systems. Within the movable EMA actuation, a step motor with a linear actuator (Misumi, Tokyo, Japan) is implemented to move the coil system along the Z-axis. The velocity of the motor is set to 0.8 mm/s. To incorporate an additional change in signal, we situate the FFP at the center of the ROI during the scanning along the Z-axis. The gradient value corresponding to the Z-axis localization is computed by matching the respective current and simulation as 1.5 T/m in the Z-direction, and 0.75 T/m in the X- and Y-directions. The mainframe of the coil system is driven forward by the connected step motor to ensure that the entire span along the Z-axis is scanned. The signals reflected by the MNPs are stored in real time until the operation of the motor is completed and it arrives at the endpoint. The highest intensity signal within each group of data is associated to its actual position within the ROI during the subsequent relocation of the peak point to scan the XY-plane. During the scanning of the XY-plane, the gradient value along the X-direction and the Y-direction is taken to be 2 T/m. During the localization scan, the target material is fixed in Rx/Tx bore. Further detailed information concerning both FFP coils and Rx/Tx coils is presented in Table 2. FFP coils are arranged in a counterclockwise sequence, with the first one being located right below the Tx/Rx coil, as depicted in Figure 4.

**Figure 5:**
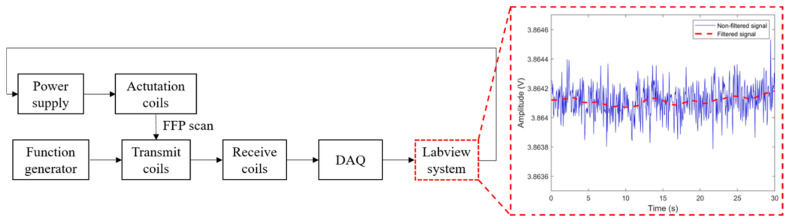
Flow chart depicting the sequence of operations in the proposed system. The signal graph on the right side shows the comparison of signal noise, depending on the filtering system in the Labview system. The peak to peak value of amplitude is 0.2 mV for the filtered case, 0.6 mV for the original case.

Finally, we assume that the injected particles filled within a phantom are expected to exhibit the highest intensity point at the center of the volume of the phantom. We continuously receive the signals induced by FFP scanning in the course of our experiments. Among other data, the maximum voltage signal extracted by the filtering system is determined to be the closest adjacent point in terms of the intensity of the nanoparticle swarm. To address the raw signals collected via FFP scanning, which include some noise arising from the hardware components, such as RF coils, we introduce digital filtering into the process of the Labview system, as illustrated in Figure 5. The widths of the basic fluctuations of the signals are reduced by using the moving average filter. The filtered signals are refined via a least-squares fitting method introduced by Savizky–Golay, thereby producing smoother data without any reduction in resolution [34].

## 4. Experiments

### 4.1. MNPs

**Figure 6:**
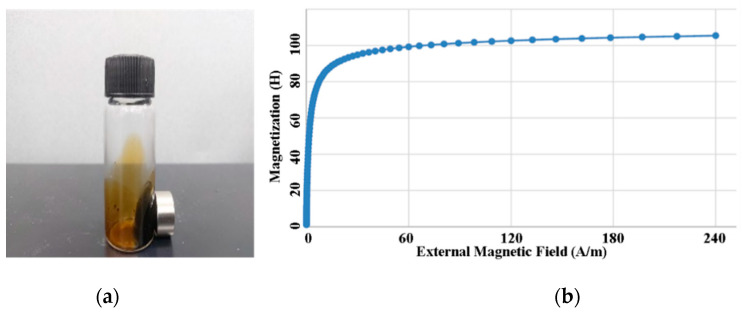
(**a**) The magnetic nanoparticle (MNP) material property (ferrofluid, EMG 707) used for scanning experiments. (**b**) The nonlinear response of magnetization of EMG 707.

Resovist (Bayer Schering Pharma AG, Leverkusen, Germany) is usually utilized as a commercial contrast agent in MRI because of its superior ability to induce high magnetic moments corresponding to particle core sizes of approximately 20 nm [35]. The other MNP candidates for MPI suffer from shortcomings due to iron cores with small diameters. Unfortunately, the production of Resovist was discontinued in 2009, and it is currently only available from I’rom Pharmaceutical (Tokyo, Japan) [12]. We faced considerable difficulty in obtaining it. However, Resovist is not the perfect MPI material for a varied distribution of particle sizes, and several MPI research groups have attempted to create alternative competitive MPI tracers [36]. Among them, we selected the water-based EMG 707 (Ferrotec, Santa Clara, CA, USA), depicted in Figure 6, as the nanoparticle tracer in this paper. This mixture comprises 1.2~6.2% magnetite ($Fe_{3}O_{4}$) particles, 7~27% water soluble dispersants, and 66.8~91.8% deionized water, which makes its average magnetic particle concentration approximately 3% by volume. The nominal particle diameter of this ferrofluid is 10 nm and its saturation point is 11 mT.

### 4.2. 2D Imaging

**Figure 7:**
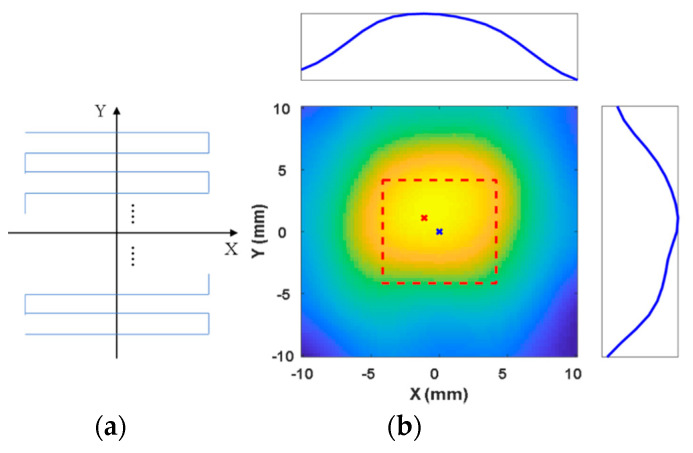
Comparison of locations of the highest intensity points of the particles and area. (**a**) The sequence of 2D scanning. (**b**) The reconstructed image based on the reflected signals.

To evaluate the basic performance of the proposed system, we conduct two-dimensional (2D) localization and imaging using an 8 × 8 × 8 ${\mathrm{mm}}^{3}$ phantom placed on the XY-plane. EMG 707 (200 μL) is injected into the phantom. The FFP is observed to pass within 1 mm of all the points within the ROI (20 × 20 ${\mathrm{mm}}^{2}$) to scan the MNPs, exposing a total of 144 points. To localize the MNPs in adequate detail, the movement of the FFP is controlled to take 1 s to move through each millimeter within the ROI with a data processing rate of 13 Hz. The sequential movement of the FFP and the experimental image are depicted in Figure 7. In the figure, the red dashed line indicates the desired region within the ROI, the blue cross denotes the expected position of the aggregation of particles, and the red cross denotes the location of the MNPs as revealed by the scan. The maximum voltage signal obtained from the filtering system is extracted as the highest intensity corresponding to the particles, as illustrated in Figure 7b. The scanned position on the 2D plane is (−1,1), indicated by the red cross in Figure 7b. The signal reflected by the MNPs (indicated by the blue line in (b)) is observed to exhibit the maximum value at the desired position (indicated by the blue cross) along the X- and Y-axes, when the FFP is generated at the position indicated by the red cross. Finally, we obtain the position of the MNPs. The root mean square error (RMSE) is measured to be 1.4 mm. Total scanning time is 144 s.

### 4.3. 3D Localization

**Figure 8:**
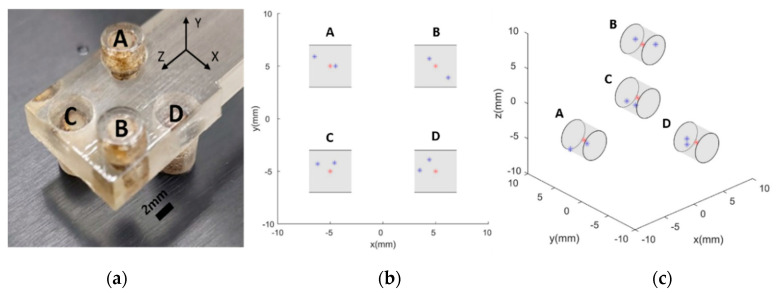
Experimentally obtained 3D localization; (**a**) phantom model, (**b**) 2D imaging on XY-plane, and (**c**) a side view of 3D imaging.

To verify the performance of the proposed imaging mechanism and quantify the achieved resolution of MNP scanning in 3D space, a simple structured phantom comprising four cylindrical holes with diameters of 4 mm filled with EMG 707 is printed using Objet Eden260VS (Stratasys, Eden Prairie, MN, USA), as illustrated in Figure 8a.

The desired intensity points corresponding to individual phantom holes in the 3D imaging experiment are taken to be (5, 5, 5), (−5, −5, 5), (5, −5, −5), and (−5, 5, −5). Because of the explicit focus on the localization of MNPs in this paper, the entire ROI was not scanned, unlike the case of conventional MPIs, which are equipped with at least three pairs of drive coils to actuate FFPs in a 3D workspace. We begin the process by identifying the position of the aggregated MNPs on the Z-axis and then proceed to scan the XY surface corresponding to the identified Z-axis position. Based on the obtained result, the 3D position of the MNPs is reconstructed. The results are presented in Figure 8b,c. The quantified results in terms of mean RMSE are observed to be 1.4, 1.4, 1.5, and 1.4 mm for the target points (A), (B), (C), and (D), respectively. The overall localization accuracy is 1.4 mm, corresponding to a single 3D phantom.

**Figure 9:**
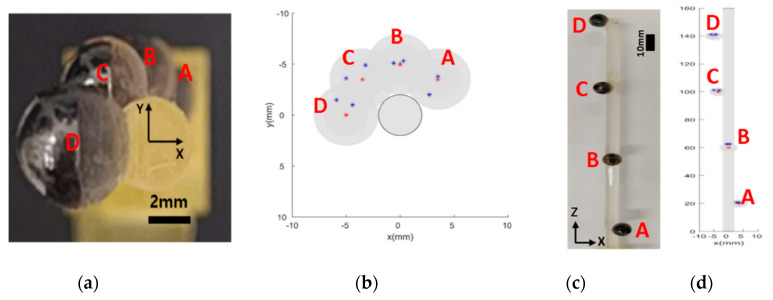
Phantom corresponding to multiple positions. The red and blue points in (**b**) and (**d**) indicate the geometric center point and the measured points of the MNP group through a 3D scan, respectively; (**a**) frontal view of the phantom, (**b**) frontal view of the 3D imaging result, (**c**) sidelong view of the phantom, and (**d**) sidelong view of the 3D imaging result.

In addition to the case of MNPs with a single fixed position, the longitudinal scanning performance of the proposed system along the axis of movement is evaluated by simultaneously using multiple groups of MNPs with multiple locations. As depicted in Figure 9, 60 µL of ferrofluid is injected into the phantom comprising four consecutive spheres on the Z-axis with distances of 40 mm between each adjacent pair. The same sequence of localization of the 3D phantom is followed in this case as well. The digital filtering system identifies the four peak points on the Z-axis scanning graph, and the motor sequentially moves back to each peak point to scan the corresponding XY-planes. The results are presented in Figure 9, where the desired positions of MNPs attached to a slender beam are detected clearly by utilizing the Z-directional movement of the prototype EMA system. Mean RMSEs corresponding to the four spheres are observed to be 0.8 mm for (A), 2.6 mm for (B), 1.9 mm for (C), and 1.8 mm for (D). A comparison of this performance with that in the case of a single swarm of particles reveals that the accuracy of multiple point scanning is slightly lower than that of the localization of a single group of MNPs. This can be attributed to the effect of adjacent particle swarms which interfere with the RF coils and the longitudinal moving mechanism.

### 4.4. Steering by Manipulating the FFP

Simultaneous imaging and actuation of MNPs using the proposed system are illustrated by using manipulating a swarm of MNPs. As mentioned previously, several existing studies have attempted the actuation of tracers using FFPs [23,24]. After locating the position of the group of MNPs and generating an FFP around it, the magnetic force generated by the FFP can be harnessed to push the particles away from it to areas with higher magnetic field intensity. This technique can be further refined if the generated magnetic field is sufficiently intense to saturate the nanoparticles, as, in this case, the effect of magnetic torque can be neglected. This enables the aligned MNPs to be actuated along a constant direction. By controlling the location of the FFP, the direction and magnitude of the force can be controlled. When the FFP is generated at the center of a coil in our system, two separate gradient field strengths are observed, as this structure is open to the outside. Thus, if nanoparticle tracers are located in front of the coil system, the tracers are one-dimensionally trapped between the two gradients, thereby enabling the proposed system to be capable of driving MNPs back and forth by controlling the step motor. Based on this intuitive concept, an experiment is conducted to validate the trapping phenomenon using ferrofluid (EMG 707) and magnetic spheres.

In this experiment, the Y-shaped phantom was scanned following the general scanning method used by the proposed system. After successful verification of the steering of MNPs in the Z-direction, a simple Y-shaped phantom was printed to validate the driving of MNPs along the X-direction by appropriately shifting the FFP position. The desired points in the phantom are selected to be (±5.5, −2, 6.3).

**Figure 10:**
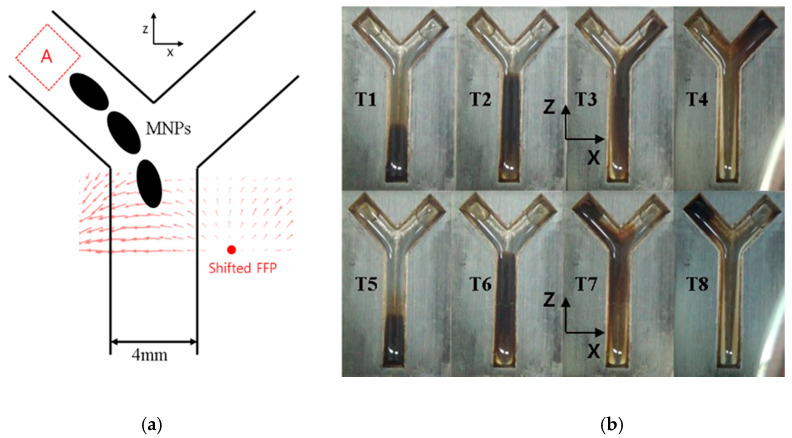
Nanoparticle manipulation to drive them to the targeted point: (**a**) schematic of steering the particles and (**b**) experimental results.

EMG 707 (10 μL) is injected into the bifurcation phantom at the initial point. The bottom of the phantom is filled with deionized water. A joystick controller (Logitech) is used to manipulate the swarm of particles in the channel. During the actuation duration required by the system, the primary structure of the coil system is observed to approach the phantom. The MNPs are forced to move along the positive Z-direction. The time frame images obtained via the experiment of the actuation are depicted in Figure 10, (T1) to (T4), and Figure 10, (T5) to (T8). At the intersection corresponding to each targeted point, the FFP is exerted on the opposite side of the desired movement direction of the nanoparticles. The time span photoshoot is depicted in Figure 10b. The upper panels of Figure 10b depict the result of steering the MNPs to the right-hand chamber of the Y channel and the lower panels present the result of steering the MNPs to the left chamber of the Y channel. The steered points are depicted in (T3) and (T4). In the case of (T3), the FFP location is steadily on the transition to -10mm. Finally, the MNPs are successfully moved to the intended position, as depicted in (T4) and (T8).

Finally, the 3D localization of the bifurcation phantom depicted in Figure 10 reveals the RSME corresponding to both desired positions (±5.5, −2, 6.3) to be 1.6 mm. Except in the case of multiple point scanning, the general resolution of 3D localization is observed to be approximately 1.5 mm.

In the experiments, the MNPs may have been expected to come to a halt perfectly at the desired positions after the drug carriers completed their movement to the destination. However, water was used as the base material of the ferrofluid, and deionized water and ferrofluid tend to become mixed together with time. Therefore, MNPs are injected into the target area of another bifurcation phantom of similar size in accordance with the assumption.

## 5. Discussions and Conclusions

In this study, we proposed a novel MNP imaging and actuation mechanism based on FFP control using a novel movable four-coil EMA system. Three types of localization experiments and an actuation experiment were conducted to evaluate its performance on 2D localization, 3D localization, and 3D localization for multiple objects. The overall mean RMSE between the measured point and the desired point was observed to be approximately 1.4 mm. In addition to imaging, both the actuation and localization of swarms of MNPs were successfully achieved using the proposed prototyped EMA system and this was validated based on Y channel experiments. The localization results based on a bifurcation phantom corroborated the feasibility of the combination of MNP actuation and MNP localization. During actuation, all particles were not perfectly moved to the target points via the manual control of FFP. However, all of them exhibited a tendency of movement in the desired direction. Additionally, a more precise FFP localization scheme with a resolution lower than 1 mm and the utilization of a magnetic particle with a bigger magnetic core size are expected to facilitate the steering of MNPs.

Compared to conventional MPI scanners that comprise multiple electromagnets with closed bore configurations [27], the proposed system reduces the number of electromagnets by utilizing a movable frame mechanism. Further, a longer workspace of approximately 400 mm was implemented, which can be further extended for particular clinical applications.

Despite the successful feasibility validation of the proposed method, further research is necessary to develop and refine similar systems. In particular, the spatial interference induced by the closed bore structure of an RF coil on an Rx/Tx coil is a critical issue in the design of a scaled-up system. Further, an advanced algorithm for FFP control and precise movement of the EMA system needs to be developed to improve the quality of both actuation and imaging. In addition, an actual image registration by incorporating X-ray image needs and safety issues related to movable parts need to be further considered to accomplish a human-scale scanner in clinical use. These advancements will be validated in animal studies in the future works.

## Figures and Tables

**Table 1:** The tendency of distance error with the desired FFP position by increasing the number of point utilized in a weighted-norm solution.

Number of Points	30	50	90	141~
Error (mm)	7	6.4	1.4	0

**Table 2:** Detailed parameters of the proposed coil system.

	Inner Diameter	Outer Diameter	Turns	Length	Wire
**Transmit coil**	0.08 mm	43.2 mm	92	40 mm	Litz wire
**Receive coil**	0.08 mm	33.6 mm	93	40 mm	Litz wire
**FFP Coil #1**	1.5 mm	62 mm	643	206 mm	copper wire
**#2**	1.5 mm	62 mm	650	206 mm	copper wire
**#3**	1.5 mm	62 mm	641	206 mm	copper wire
**#4**	1.5 mm	62 mm	671	206 mm	copper wire
